# Nuclear PD-L1: an emerging oncogenic driver and promising therapeutic target in cancer

**DOI:** 10.1186/s12929-026-01268-5

**Published:** 2026-06-17

**Authors:** Citing Guo, Zenan Fan, Cuiyan Guo, Yaning Yang, Helei Hou, Dantong Sun

**Affiliations:** 1 Key Laboratory of Carcinogenesis and Translational Research (Ministry of Education/Beijing), Department of Genitourinary Oncology, Peking University Cancer Hospital and Institute, Beijing, 100142 China; 2 Department of Medical Oncology, Peking University First Hospital, Peking University, Beijing, 100034 China; 3 Department of Respiratory and Critical Care Medicine, Peking University First Hospital, Beijing, 100034 China; 4 Centre de Recherche des Cordeliers, Equipe Labellisée par la Ligue Contre le Cancer, Inserm U1138, Université Paris Cité, Sorbonne Université, 75006 Paris, France; 5 Metabolomics and Cell Biology Platforms, Gustave Roussy Institut, 94805 Villejuif, France; 6 Department of Oncology, The Affiliated Hospital of Qingdao University, Qingdao University, Qingdao, 266000 Shandong China

**Keywords:** Nuclear PD-L1, Nuclear translocation, Post-translational modification, Therapeutic resistance, Cancer adaptation

## Abstract

Programmed cell death-ligand 1 (PD-L1) is best known as a membrane immune checkpoint; however, accumulating evidence indicates that PD-L1 can also localize to the nucleus, where it may exert PD-1-independent, cell-intrinsic functions in cancer. Emerging studies associate nuclear PD-L1 (nPD-L1) with aggressive disease, therapeutic resistance, and poor outcomes across multiple malignancies. In this review, we summarize current evidence regarding the regulatory mechanisms that may govern PD-L1 nuclear translocation, including post-translational modifications, stress-responsive signaling, and importin-dependent trafficking. We further discuss how nPD-L1 has been linked to adaptive programs involving DNA damage repair, metabolic rewiring, transcriptional regulation, and tumor microenvironment remodeling in context-dependent models. Clinically, nPD-L1 has potential relevance as a prognostic biomarker and as a candidate indicator of resistance to immunotherapy, radiotherapy, and chemotherapy, although prospective validation remains limited. We also highlight current challenges in detection and quantification, including the need for standardized multiplex imaging and digital pathology approaches. Finally, we discuss emerging therapeutic strategies aimed at disrupting PD-L1 nuclear trafficking or selectively targeting nuclear PD-L1-associated functions. Collectively, these findings support nPD-L1 as an important and potentially actionable dimension of PD-L1 biology that warrants further mechanistic and translational investigation.

**Graphical Abstract:**

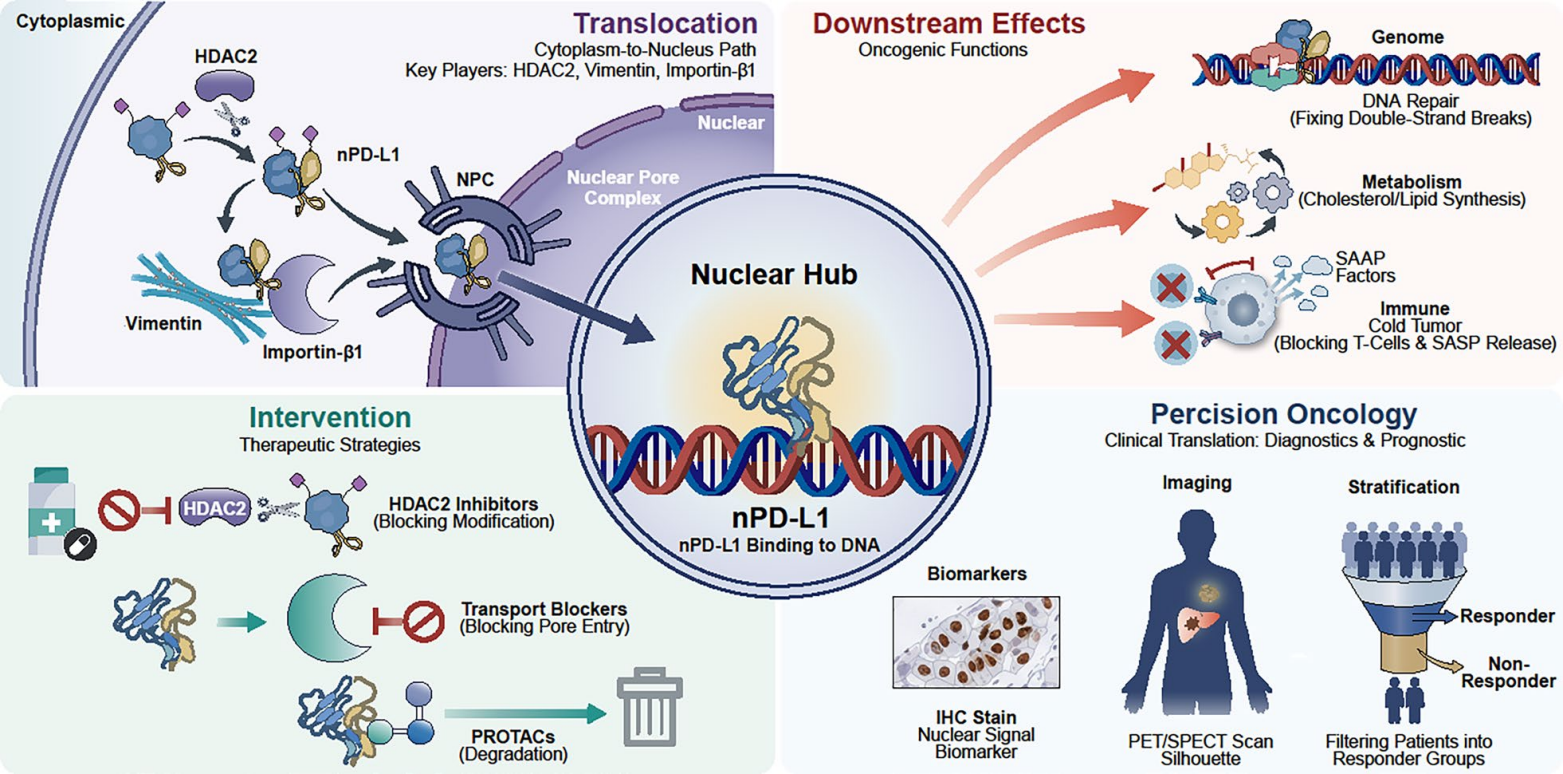

## Introduction

Programmed cell death-ligand 1 (PD-L1) is canonically known as a membrane-bound immune checkpoint, suppressing T-cell activity via PD-1 interaction to facilitate tumor immune escape, which serves as a mechanism successfully targeted by immunotherapy [1–3]. However, the stark heterogeneity in patient response and prevalent acquired resistance underscore a more complex biology [4, 5]. Interestingly, the identification of nuclear PD-L1 (nPD-L1) has broadened the current view of PD-L1 biology, suggesting functions beyond its established role as a membrane immune checkpoint ligand. Accumulating evidence indicates that PD-L1 may also exert PD-1-independent, cell-intrinsic activities within the nucleus [6–10]. Elevated nPD-L1 expression has been associated with aggressive disease, therapeutic resistance, and poor survival across multiple cancer types [11–13]. Mechanistically, nPD-L1 has been implicated in several adaptive nuclear programs, including DNA repair, metabolic rewiring, and tumor microenvironment remodeling [14–16].

However, several challenges continue to limit mechanistic integration and clinical translation. Reported regulators of PD-L1 nuclear translocation remain fragmented across diverse and often context-specific model systems, while its nuclear functions are frequently described in isolation rather than within a more integrated framework [7, 17, 18]. In addition, standardized detection strategies for nPD-L1 are still lacking. To address these gaps, this review synthesizes current evidence into an integrated translocation-to-function framework and introduces a Post-translational modification (PTM) dual-gate model as a working hypothesis to help explain how coordinated trafficking checkpoints may regulate PD-L1 nuclear import. We further discuss how these mechanistic insights may inform biomarker development and therapeutic intervention in refractory cancers.

### From membrane to nucleus: the dynamic regulatory pathways of PD-L1 nuclear translocation

**Fig. 1:**
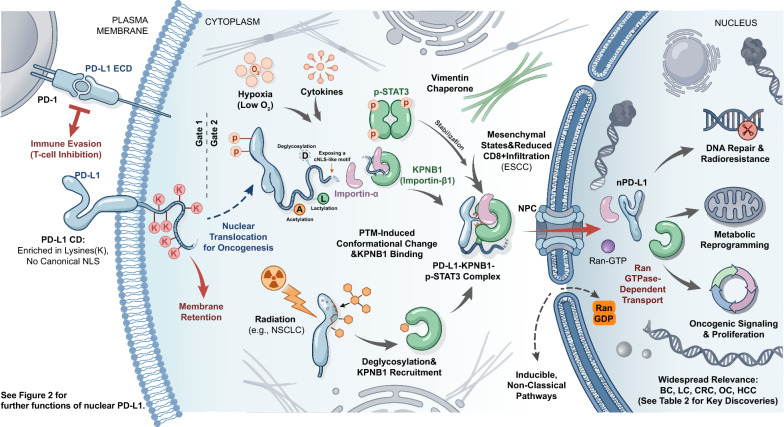
Context-dependent nuclear translocation of PD-L1 and its oncogenic functions in cancer. Under basal conditions, PD-L1 is retained at the plasma membrane, where it suppresses T-cell activity through PD-1 engagement. In response to cellular stress, post-translational modifications are proposed to promote PD-L1 nuclear trafficking through an importin-α/β1-mediated pathway, followed by RanGTP-dependent cargo release in the nucleus. Nuclear PD-L1 has been associated with DNA repair, metabolic adaptation, inflammation, and therapy resistance across multiple tumor contexts. This figure summarizes an integrated translocation-to-function framework based on observations from diverse model systems and should be considered a working model that remains to be fully validated

**Fig. 2:**
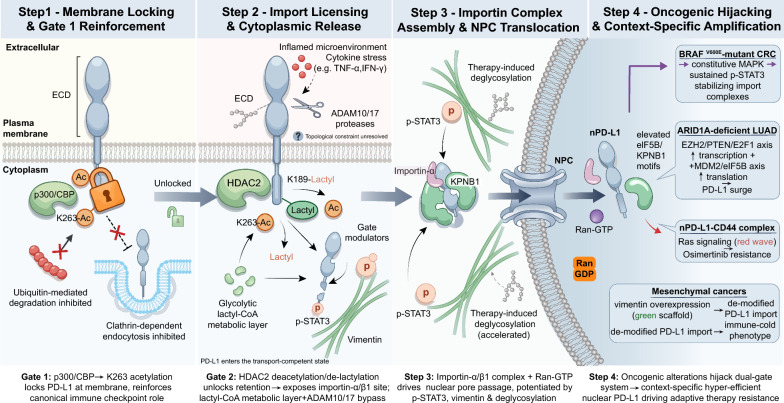
PTM-gated model controlling PD-L1 nuclear translocation and oncogenic pathway hijacking. Sequential regulatory steps may govern PD-L1 trafficking, including membrane retention, import licensing, nuclear transport, and context-specific signaling outputs. Acetylation, deacetylation, lactylation, and deglycosylation are candidate post-translational checkpoints that may influence transport competence. In selected tumor types, oncogenic pathways may further amplify this process and shape downstream functional outputs. This schematic represents a working hypothesis that integrates multiple potential trafficking routes and remains to be fully validated

How PD-L1 transitions from a membrane immune checkpoint ligand to a nuclear effector remains a central question. PD-L1 is a type I transmembrane glycoprotein whose localization determines function [14, 19]: membrane PD-L1 suppresses antitumor immunity, whereas nuclear PD-L1 has been linked to DNA repair, metabolic adaptation, proliferation, and therapeutic resistance (as displayed in Table 1, Figs. 1 and  2).

**Table 1:** Comparison of canonical membrane PD-L1 and reported nuclear PD-L1 functions in cancer

Aspect	Membrane PD-L1 (Classical)	Nuclear PD-L1*
Subcellular localization & detection	Primarily localizes to the cell surface. Detected by IHC, flow cytometry, or IF (membranous staining); membrane fractionation/WB	Primarily localizes within the nucleus. Detected by nuclear fractionation/WB, IF/confocal microscopy (nuclear staining), or IHC with nuclear-specific scoring; requires deglycosylation (e.g., PNGase F) for confirmation
Representative reported functions	Immune checkpoint inhibition; suppresses T-cell activation via PD-1/PD-L1 interaction	Transcriptional regulation; promotes intrinsic tumor cell functions (proliferation, DNA repair, inflammation) independent of PD-1 immune checkpoint
Key downstream pathways & effects	PD-1 signaling inhibits TCR pathway, reduces cytokine production, promotes T-cell exhaustion/apoptosis	Activates NF-κB/CGAS–STING (inflammation/SASP), ATR/Chk1 (DSB repair), STAT3/EGR1 (angiogenesis); enhances proliferation via GAS6/MerTK; upregulates SQLE (cholesterol synthesis); switches apoptosis to pyroptosis via GSDMC
Impact on tumor cells	Enhances survival & resistance to apoptosis; promotes EMT and metastasis; stabilized via glycosylation/ubiquitination	Accelerates proliferation & invasion; enhances DNA repair; regulates cell cycle; induces senescence/pyroptosis; confers chemoresistance (e.g., via EZH2/PTEN/E2F1)
Interaction with tumor microenvironment	Suppresses CD8 + T and NK cells; recruits immunosuppressive cells (MDSCs, Tregs); reduces TIL infiltration	Modulates SASP/cytokines (e.g., IL-6, IFN-**γ**); inversely correlates with CD8 + T-cell infiltration; promotes CAF/MDSC recruitment; enhances immune evasion via transcriptional reprogramming
Clinical significance & therapeutic implications	High expression associated with poor OS/PFS; predictive biomarker for anti-PD-1/PD-L1 ICB response; targeted by monoclonal antibodies (e.g., atezolizumab)	High expression correlates with poor prognosis, recurrence, and chemoresistance (e.g., in ovarian, breast, colorectal cancers). Emerging target: HDAC2 inhibitors (e.g., Santacruzamate A) to block nuclear translocation; potential for combination with ICB, RT, or chemotherapy; prognostic biomarker in resistant cohorts

*IHC* Immunohistochemistry, *IF* Immunofluorescence, *WB* Western Blot, *TCR* T-cell Receptor, *NF-κB* Nuclear Factor kappa B, *CGAS–STING* Cyclic GMP-AMP Synthase–Stimulator of Interferon Genes, *SASP* Senescence-Associated Secretory Phenotype, *ATR/Chk1* Ataxia Telangiectasia and Rad3-related Protein/Checkpoint Kinase 1, *DSB* Double-Strand Break, *STAT3/EGR1* Signal Transducer and Activator of Transcription 3/Early Growth Response 1, *GAS6/MerTK* Growth Arrest-Specific 6/Mer Tyrosine Kinase, *SQLE* Squalene Epoxidase, *GSDMC* Gasdermin C, *EMT* Epithelial-Mesenchymal Transition, *EZH2/PTEN/E2F1* Enhancer of Zeste Homolog 2/Phosphatase and Tensin Homolog/E2F Transcription Factor 1, *IL-6* Interleukin-6, *IFN-γ* Interferon Gamma, *CAF* Cancer-Associated Fibroblast, *MDSC* Myeloid-Derived Suppressor Cell, *TIL* Tumor-Infiltrating Lymphocyte, *OS* Overall Survival, *PFS* Progression-Free Survival, *ICB* Immune Checkpoint Blockade, *RT* Radiotherapy, *HDAC2* Histone Deacetylase 2

^*^Reported nuclear PD-L1 functions remain context-dependent and incompletely validated

Structurally, PD-L1 contains a short cytoplasmic tail enriched in modifiable lysine residues and lacking a canonical nuclear localization signal (NLS) [7]. Nevertheless, the intrinsically disordered nature of this cytoplasmic tail is thought to functionally mimic a classical NLS (cNLS), enabling recognition by Importin-α and subsequent recruitment of Importin-β to facilitate nuclear translocation. Consequently, nuclear entry appears unlikely to be constitutive, but rather depends on inducible trafficking pathways that can be activated by cellular stressors, including hypoxia, inflammatory signaling, chemotherapy, and radiotherapy [20–22]. Once inside the nucleus, RanGTP-driven dissociation of the cargo-importin complex releases PD-L1 to exert its nuclear functions. Nuclear PD-L1 has now been reported across multiple tumor types, including breast, lung, colorectal, ovarian and hepatocellular cancers, supporting the broad relevance of this pathway (Tables 2 and  3) [6–16, 23–38].

**Table 2:** Studies reporting nPD-L1 expression and its clinical and functional significance

Study	Publication year	Detection method	Cancer type	Key findings	Proposed mechanism	Potential translational relevance
Ghebeh et al.	2010	IHC, IF, WB	BC	Doxorubicin downregulates cell surface PD-L1 and upregulates nPD-L1; nPD-L1 inhibits apoptosis	Doxorubicin induces PD-L1 nuclear translocation via p-AKT; nPD-L1 promotes anti-apoptotic effects	Supports combining cytotoxic chemotherapy with strategies that limit PD-L1 nuclear redistribution
Chen et al.	2014	IHC, TMA, IF, WB	EC	Membrane/cytoplasm PD-L1 correlates with tumor invasion and poor OS; nPD-L1 correlates with invasion but not OS	PD-L1 expression in membrane/cytoplasm and nucleus differentially regulates tumor invasion	Suggests PD-L1 subcellular localization may refine biomarker interpretation in EC
Satelli et al.	2016	IF, WB, Confocal microscopy	CRC, PC	nPD-L1 in CSV-positive CTCs associates with poor PFS and OS	nPD-L1 in EMT-CTCs promotes tumor progression	Supports evaluation of nPD-L1-positive CTCs as a liquid-biopsy biomarker for risk stratification
Parra et al.	2019	IHC	LADC, LSCC, MM, RCC, HCC, DBC	PD-L1 shows membrane, cytoplasmic, and nuclear patterns in tumor and non-tumor cells	Variable PD-L1 subcellular localization affects IHC interpretation	Highlights the need for localization-aware PD-L1 pathology scoring
Schulz et al.	2020	WB, FACS, IF, IP	HNSCC	nPD-L1 interacts with AKT1 post-IR, promoting NHEJ DSB repair and radioresistance	PD-L1 nuclear translocation post-IR enhances Ku binding to DSBs	Suggests targeting nPD-L1-associated DNA repair pathways to enhance radiosensitivity
Hou et al.	2020	WB, IF, Cellular fractionation, IHC	BC (mainly TNBC), multiple	Hypoxia induces nPD-L1, upregulating GSDMC; caspase-8 cleaves GSDMC causing pyroptosis and tumor necrosis; correlates with poor survival	p-Stat3 mediates PD-L1 nuclear translocation; nPD-L1/p-Stat3 transcribes GSDMC; TNF**α** activates caspase-8 for cleavage	Suggests vulnerability of the nPD-L1/GSDMC axis in selected breast cancer settings
Yu et al.	2020	IP, WB, IF, FISH, Cell fractionation	TNBC, PC, CRC, LC	PD-L1 depletion suppresses proliferation, colony formation, and tumor growth independent of PD-1; leads to multinucleated cells and cohesion defects	nPD-L1 in cohesin complex compensates for Sororin loss, competes with WAPL for PDS5B binding, regulating chromatid cohesion	Supports exploring nPD-L1-dependent cohesion pathways in tumors with replication stress
Gao et al.	2020	WB, IF, IP, ChIP-seq	BC	Acetylation by p300 promotes nPD-L1, regulating immune genes	HDAC2 deacetylation facilitates nPD-L1 translocation	Supports modulation of PD-L1 acetylation/nuclear trafficking to improve checkpoint blockade response
Shim et al.	2021	IHC	PC	Membrane and nPD-L1 correlate with Gleason score; no prognostic value for BCR	nPD-L1 expression linked to tumor invasion	Suggests further evaluation of nPD-L1 as a biomarker in localized prostate cancer
Ma et al.	2022	WB, IF, IHC, ChIP-seq	CRC	nPD-L1 binds LEF-1 to promote cell cycle via BUB1 upregulation	p-ERK facilitates nPD-L1 translocation	Suggests targeting nPD-L1-linked proliferative signaling in BRAF-mutant CRC
Du et al.	2021	WB, IF, IP, ChIP	NSCLC	nPD-L1 binds Sp1 to upregulate Gas6, activating MerTK and proliferation	Vimentin promotes nPD-L1 translocation	Supports combined inhibition of PD-L1 trafficking and Gas6/MerTK signaling in NSCLC
Lee et al.	2024	WB, IF, ChIP	Melanoma, CRC, LC	nPD-L1 depletion induces senescence via STING upregulation	nPD-L1 binds STING promoter, repressing transcription	Suggests therapeutic induction of senescence through nPD-L1 pathway disruption
Yu et al	2023	WB, IF, IHC, IP	UM	nPD-L1 interacts with p-STAT3 to activate EGR1, promoting angiogenesis	HDAC2 deacetylation facilitates nPD-L1 translocation	Supports evaluating HDAC2/nPD-L1 signaling as an anti-angiogenic strategy in UM
Romeo et al.	2023	WB, IF, IP	PC	PD-L1 acetylation regulates nuclear translocation; interacts with TRAPPC4 for recycling	Acetylation-dependent nPD-L1 promotes proliferation	Suggests PD-L1 acetylation status may represent a therapeutic vulnerability in PC
Schulz et al.	2024	WB, IF, LC–MS, PLA	HNC	nPD-L1 variants regulate stemness via vimentin interaction	nPD-L1 promotes CSC properties	Supports investigating nPD-L1-related stemness pathways in head and neck cancer
Liang et al	2024	IHC, IF, WB	ESCC	Vimentin promotes nPD-L1, inversely correlating with CD8 + T infiltration	Vimentin interacts with PD-L1 for nuclear translocation	Suggests targeting vimentin-associated trafficking may enhance immunotherapy responsiveness in ESCC
Kim et al.	2023	WB, IF, IHC	BC	BRD4 promotes nPD-L1, which upregulates RelB and IL-6 for stemness	BRD4/nPD-L1/RelB axis drives BCSC formation	Supports evaluation of BRD4/nPD-L1 signaling in breast cancer stem-like states
Shu et al.	2024	WB, IF, IP	NSCLC	Deglycosylation promotes nPD-L1, enhancing Ku binding and NHEJ	CMTM6-dependent deglycosylation facilitates nPD-L1 translocation	Suggests inhibition of nPD-L1 trafficking may improve radiotherapy response in NSCLC
Wu et al.	2024	WB, IF, CRISPR	CRC	NUP43 promotes nPD-L1, activating TM4SF1/JAK/STAT3 and PD-L1 feedback	nPD-L1 upregulates TM4SF1 for JAK/STAT3 activation	Supports targeting the NUP43–nPD-L1 signaling axis in CRC
Liu et al.	2024	WB, IF, IHC	Multiple (HNSCC, BC, etc.)	LA induces nPD-L1, phosphorylating mH2A1 via p-AMPKα, activating senescence genes	nPD-L1/mH2A1 interaction promotes senescence and overcomes ICB resistance	Suggests context-dependent therapeutic exploitation of senescence-associated nPD-L1 signaling
Qin et al.	2024	Bioinformatics (TCGA, GEO)	LC	RPA1-ETAA1 axis promotes nPD-L1, reshaping TIME	RPA1-dependent ETAA1 facilitates nPD-L1 translocation	Supports further validation of the RPA1–ETAA1 axis as a candidate therapeutic target in lung cancer
Lu et al.	2025	WB, IF, IHC, PET	CC	NAD + metabolism via NAMPT/SIRT1 promotes nPD-L1, regulating immune genes	SIRT1 deacetylation facilitates nPD-L1 translocation	Suggests targeting NAD + /SIRT1-dependent PD-L1 trafficking to augment immunotherapy in CC
Nihira et al.	2025	WB, IF, IP	TNBC	nPD-L1 activates ATR/Chk1 and cGAS-STING, promoting inflammation	ATR/Chk1 inhibitors block nPD-L1 effects	Supports combining checkpoint blockade with ATR/Chk1 pathway inhibition in relevant contexts
Wang et al.	2025	scRNA-seq, WB, IF, RNA-seq, ChIP-seq, Co-IP	TNBC	IFN-γ enhances metastasis via nPD-L1; PD-L1-POLR2A upregulates LY6E	IFN-γ induces HDAC2 deacetylation of PD-L1 for nuclear entry; nPD-L1-POLR2A complex activates LY6E promoter driving metastasis	Suggests disrupting nPD-L1 transcriptional complexes may limit metastasis in TNBC
Sun et al.	2025	Spatial transcriptomics, WB, IP	LUAD	ARID1A deficiency promotes nPD-L1 via MDM2/eIF5B, activating RAS	nPD-L1/CD44 complex activates RASGEF1A	Supports multi-level targeting of nPD-L1 biogenesis/trafficking in ARID1A-deficient LUAD
Wang et al.	2025	WB, IF, IP	LC	Delactylation by HDAC2 promotes nPD-L1 via vimentin, upregulating SQLE	p300 lactylates PD-L1; delactylation enhances nuclear translocation	Suggests metabolic targeting of the HDAC2–nPD-L1–SQLE axis in liver cancer
Zhu et al.	2025	WB, IF, Flow cytometry	NSCLC	NAT10-mediated ac4C on KPNB1 promotes nPD-L1, inducing immune escape	ac4C modification stabilizes KPNB1 for PD-L1 nuclear import	Supports NAT10/KPNB1 pathway inhibition as a radiosensitization strategy in NSCLC
Asare-Werehene et al.	2025	IF, Digital image analysis	OVCA	Increased nPD-L1 associated with recurrence, chemoresistance, poor survival; hinders CD8 + T cell benefits; co-expression with pGSN worsens outcomes	nPD-L1 interacts with pGSN, suppressing CD8 + T cells in TME, promoting chemoresistance	Suggests nPD-L1 may help identify chemoresistant OVCA subsets for combination therapy development

*IHC* Immunohistochemistry, *IF* Immunofluorescence, *WB* Western Blot, *BC* Breast Cancer, *OS* Overall Survival, *EC *Endometrial Cancer, *TMA* Tissue Microarray, *CRC* Colorectal Cancer, *PC* Prostate Cancer, *CSV* Cell Surface Vimentin, *CTCs* Circulating Tumor Cells, *PFS* Progression-Free Survival, *LADC* Lung Adenocarcinoma, *LSCC* Lung Squamous Cell Carcinoma, *MM* Malignant Melanoma, *RCC* Renal Cell Carcinoma, *HCC* Hepatocellular Carcinoma, *DBC* Ductal Breast Carcinoma, *FACS* Fluorescence-Activated Cell Sorting, *IP* Immunoprecipitation, *HNSCC* Head and Neck Squamous Cell Carcinoma, *IR* Ionizing Radiation, *NHEJ* Non-Homologous End Joining, *DSB* Double-Strand Break, *TNBC* Triple-Negative Breast Cancer, *GSDMC* Gasdermin C, *FISH* Fluorescence In Situ Hybridization, *LC* Lung Cancer, *BCR* Biochemical Recurrence, *ChIP-seq* Chromatin Immunoprecipitation Sequencing, *LEF-1* Lymphoid Enhancer-Binding Factor 1, *BUB1* Budding Uninhibited by Benzimidazoles 1, *p-ERK* phosphorylated Extracellular Signal-Regulated Kinase, *NSCLC* Non-Small Cell Lung Cancer, *Sp1* Specificity Protein 1, *Gas6* Growth Arrest-Specific 6, *MerTK* MER Proto-Oncogene Tyrosine Kinase, *STING* Stimulator of Interferon Genes, *UM* Uveal Melanoma, *p-STAT3* phosphorylated Signal Transducer and Activator of Transcription 3, *EGR1* Early Growth Response 1, *HDAC2* Histone Deacetylase 2, *TRAPPC4* Trafficking Protein Particle Complex Subunit 4, *HNC* Head and Neck Cancer, *LC–MS* Liquid Chromatography-Mass Spectrometry, *PLA* Proximity Ligation Assay, *CSC* Cancer Stem Cell, *ESCC* Esophageal Squamous Cell Carcinoma, *BRD4* Bromodomain-Containing Protein 4, *RelB* RelB Proto-Oncogene, NF-KB Subunit, *IL-6* Interleukin-6, *BCSC* Breast Cancer Stem Cell, *CMTM6* CKLF Like MARVEL Transmembrane Domain Containing 6, *NUP43* Nucleoporin 43, *TM4SF1* Transmembrane 4 L Six Family Member 1, *JAK* Janus Kinase, *STAT3* Signal Transducer and Activator of Transcription 3, *LA* Lactic Acid, *mH2A1* macroH2A1, *p-AMPKα* phosphorylated AMP-Activated Protein Kinase alpha, *ICB* Immune Checkpoint Blockade, *TCGA* The Cancer Genome Atlas, *GEO* Gene Expression Omnibus, *RPA1* Replication Protein A1, *ETAA1* Ewing Tumor Associated Antigen 1, *TIME* Tumor Immune Microenvironment, *p300* E1A Binding Protein P300, *CC* Cervical Cancer, *NAD + * Nicotinamide Adenine Dinucleotide, *NAMPT* Nicotinamide Phosphoribosyltransferase, *SIRT1* Sirtuin 1, *ATR* Ataxia Telangiectasia and Rad3-Related, *Chk1* Checkpoint Kinase 1, *cGAS* Cyclic GMP-AMP Synthase, *scRNA-seq* Single-Cell RNA Sequencing, *RNA-seq* RNA Sequencing, *Co-IP* Co-Immunoprecipitation, *IFN-γ* Interferon-gamma, *POLR2A* RNA Polymerase II Subunit A, *LY6E* Lymphocyte Antigen 6 Family Member E, *ARID1A* AT-Rich Interaction Domain 1A, *MDM2* Mouse Double Minute 2 Homolog, *eIF5B* Eukaryotic Translation Initiation Factor 5B, *RAS* Rat Sarcoma Virus, *LUAD* Lung Adenocarcinoma, *RASGEF1A* Ras Guanine Nucleotide Exchange Factor 1A, *SQLE* Squalene Epoxidase, *NAT10* N-Acetyltransferase 10, *ac4C* N4-Acetylcytidine, *KPNB1* Karyopherin Subunit Beta 1, *RT* Radiotherapy, *OVCA* Ovarian Cancer, *pGSN* Plasma Gelsolin, *TME* Tumor Microenvironment

**Table 3:** Clinical studies evaluating associations between nPD-L1 and patient outcomes

Study	Year	Cancer type	Cohort size (n)	Detection method for nPD-L1	Survival association reported	Independent risk factor (MVA)	Treatment context
Chen et al.	2014	EC	95 human EC tissues (TMA); cell lines	IHC on TMA (clone E1L3N; membranous/cytoplasmic and nuclear staining)	Membrane/cytoplasm positive: poor OS (HR = 2.157 [1.017–4.577], p = 0.0452); nuclear positive: assoc. tumor invasion (p = 0.0331), no sig. OS diff. (p = 0.6755)	NA	Surgical resection
Satelli et al.	2016	CRC, PC	62 mCRC patients, 30 mPC patients (peripheral blood for CTCs)	IF on isolated CTCs (CSV mAb magnetic separation; confocal microscopy)	nPD-L1 + : short survival (p < 0.05); poor OS in CRC, poor PFS in PC	NA	Undergoing treatment (unspecified)
Shim et al.	2021	PC	172 PC patients (needle biopsy tissues)	IHC on biopsy cores (clone unspecified; membranous and nuclear scoring)	nPD-L1 not predictive of BCR-free survival (p = 0.6755)	No	Primary RT
Ma et al.	2022	CRC	CRC tissues (unspecified cohort size)	IHC (clone unspecified)	nPD-L1 assoc. poor OS (p < 0.05)	NA	NA
Liang et al	2024	ESCC	ESCC tissues (TMA via mIF)	mIF (multiplex immunofluorescence; anti-PD-L1 antibody)	nPD-L1 assoc. recurrence and poor OS (p < 0.05)	NA	NA
Kim et al.	2023	BC	BC tissues/cell lines/mouse models	IHC (clone unspecified); WB/IF for confirmation	nPD-L1 assoc. poor OS (p < 0.05)	Yes	Chemo
Yu et al	2023	UM	UM samples (tissues); cell lines	IHC (clone unspecified)	nPD-L1 assoc. unfavorable outcome (p < 0.05)	NA	NA
Hou et al	2020	BC, CRC	Various cancer tissues/cell lines/mouse models	IHC (clone unspecified)	nPD-L1 assoc. poor OS (p < 0.05)	NA	ICB
Asare-Werehene et al	2025	OVCA	OVCA samples (tissues)	IF (immunofluorescence; anti-PD-L1 antibody)	nPD-L1 assoc. poor OS/DFS (p < 0.05); assoc. chemoresistance	NA	Chemo

*EC* Esophageal Cancer, *CRC* Colorectal Cancer, *PC* Prostate Cancer, *ESCC* Esophageal Squamous Cell Carcinoma, *BC* Breast Cancer, *UM* Uveal Melanoma, *OVCA* Ovarian Cancer, *TMA* Tissue Microarray, *IHC* Immunohistochemistry, *IF* Immunofluorescence, *mIF* Multiplex Immunofluorescence, *WB* Western Blot, *CTCs* Circulating Tumor Cells, *CSV* Cell Surface Vimentin, *mAb* Monoclonal Antibody, *nPD-L1* Nuclear Programmed Death-Ligand 1, *OS* Overall Survival, *PFS* Progression-Free Survival, *DFS* Disease-Free Survival, *BCR* Biochemical Recurrence, *HR* Hazard Ratio, *CI* Confidence Interval, *MVA* Multivariate Analysis, *NA* Not Assessed/Not Available, *RT* Radiotherapy, *ICB* Immune Checkpoint Blockade, *Chemo* Chemotherapy

A key unresolved issue concerns the molecular origin of nPD-L1. Here it is important to distinguish two conceptually separate questions that operate at different levels of the pathway: (i) how PD-L1 is brought into a state where it is exposed to the cytoplasm and competent for transport (the sourcing step), and (ii) how it subsequently crosses the nuclear envelope (the translocation step). With respect to sourcing, several non–mutually-exclusive routes have been proposed. The prevailing model suggests that plasma membrane-localized PD-L1 undergoes endocytosis, followed by retrograde intracellular trafficking and subsequent nuclear import [38–40]. This route conceptually couples loss of surface immune checkpoint activity with gain of nuclear oncogenic functions. However, alternatively, PD-L1 may be rerouted intracellularly from the endoplasmic reticulum or Golgi apparatus before reaching the plasma membrane, or undergo proteolytic processing to generate cytoplasmic fragments capable of nuclear translocation [41, 42]. Current biochemical evidence, including detection of high-molecular-weight nuclear species, favours full-length or heavily modified PD-L1 as a predominant nuclear form, although the relative contribution of cleaved intermediates remains uncertain [35, 39].

Regardless of how this cytoplasmic pool is generated, these upstream routes are thought to converge on a single shared downstream mechanism—the canonical importin-dependent transport system. Available data support a model in which PD-L1 co-opts the canonical importin-dependent transport system after stimulus-induced remodeling of its cytoplasmic domain [7, 21, 43, 44]. PTMs may expose lysine-rich cNLS-like motifs in the PD-L1 cytoplasmic tail, enabling recognition by Importin-α; Importin-α in turn recruits Importin-β1 through its importin-β-binding (IBB) domain to assemble the classical trimeric import complex that facilitates translocation through the nuclear pore complex [45, 46]. Additional cofactors, including p-STAT3, vimentin and therapy-induced deglycosylation, may enhance transport efficiency in a context-dependent manner [47, 48]. Rather than acting as universal transport factors, these proteins are more likely to function as permissive scaffolds or signal amplifiers that increase the probability of nuclear import under specific oncogenic or stress conditions [14, 49]. To integrate these observations, we propose a PTM-gated trafficking framework consisting of four sequential regulatory steps.

### Step 1: membrane retention switch

A proposed stepwise gating framework provides a tentative mechanistic explanation for PD-L1 nuclear translocation, primarily based on observations in specific cancer cell lines like MDA-MB-231 [6, 14, 39]. However, its generalizability across diverse tumor types and physiological stress conditions remains to be fully validated [50]. Gate 1 (Membrane Retention): In the model, acetylation at lysine 263 (K263), catalyzed by p300/CBP, functions as the first regulatory switch governing PD-L1 membrane residency. In its acetylated state, K263 stabilizes PD-L1 at the plasma membrane by competing with ubiquitin-mediated degradation and sterically blocking AP2 recognition, thereby reinforcing its canonical immune-inhibitory role [51–55]. Conversely, histone deacetylase 2 (HDAC2)-mediated deacetylation restores cytoplasmic tail accessibility to the HIP1R-AP2 endocytic machinery, permitting clathrin-mediated internalization as the first committed step toward nuclear translocation [7, 56]. Thus, the K263 acetylation/deacetylation cycle function as a membrane-retention switch that couples surface residency to endocytic sorting. The key regulators at this gate-p300/CBP (negative regulator), HDAC2 (positive regulator), and acetylation at K263 (trafficking switch)-are summarized in Table 4 (as shown in Fig. 1, Gate 1; Fig. 2, Step 1).

**Table 4:** Reported regulators of PD-L1 nuclear translocation: proposed mechanisms and representative cancer contexts

Regulator	Category	Proposed role in PD-L1 nuclear translocation	Representative mechanism described in literature	Representative cancer context(s)
HDAC2	Deacetylase	Positive regulator	Deacetylation of PD-L1 (K263) and/or delactylation (K189) has been reported to facilitate nuclear localization of PD-L1	BC, HCC, UM
p300/CBP	Acetyltransferase	Negative regulator of nuclear trafficking	Acetylation of PD-L1 at K263 has been associated with membrane retention and reduced nuclear translocation	BC
Importin-α (KPNA family)	Nuclear transport adaptor	Transport mediator	Recognizes cargo proteins carrying classical NLS-like motifs and may participate in PD-L1 nuclear import	NSCLC, BC
Importin-β1 (KPNB1)	Nuclear import receptor	Core transport mediator	Mediates translocation of cargo complexes through the nuclear pore complex; reported to support PD-L1 nuclear import	NSCLC, CC
RanGTP	Small GTPase	Directionality factor	Canonical mediator of cargo release from importins after nuclear entry; inferred to participate in PD-L1 import cycles	Pan-cancer*
p-STAT3	Signaling cofactor	Context-dependent facilitator	May associate with PD-L1-containing complexes and enhance nuclear trafficking and transcriptional activity	BC, UM
Vimentin	Cytoskeletal protein	Context-dependent facilitator	Reported to interact with PD-L1 and promote perinuclear trafficking/nuclear localization	NSCLC, ESCC, TNBC
ADAM10/17	Metalloproteases	Candidate upstream regulator	Cleavage of membrane PD-L1 may alter PD-L1 trafficking dynamics and availability for intracellular redistribution	Multiple*
eIF5B	Translation factor	Upstream expression regulator	Enhances PD-L1 protein translation and may indirectly increase the pool available for nuclear translocation	LUAD
NAT10	RNA acetyltransferase	Indirect positive regulator	ac4C modification of KPNB1 mRNA increases KPNB1 expression, thereby supporting PD-L1 nuclear import	NSCLC
CMTM6	PD-L1 stability regulator	Context-dependent regulator	Stabilizes PD-L1 protein; altered glycosylation/stability has been linked to nuclear redistribution in some models	NSCLC
p-AKT	Stress-responsive kinase	Positive regulator	Genotoxic stress-associated AKT activation has been linked to PD-L1 nuclear redistribution	BC, HNSCC
ERK signaling	MAPK pathway	Positive regulator	ERK activation has been associated with increased PD-L1 nuclear localization in BRAF-driven models	CRC
N-glycosylation (N219)	Post-translational modification	Negative regulator of nuclear trafficking	Glycosylated PD-L1 is preferentially retained at the membrane; deglycosylation may facilitate nuclear entry	NSCLC
Lactylation (K189)	Post-translational modification	Context-dependent regulator	Lactylation status of PD-L1 has been linked to regulation of its nuclear localization in liver cancer models	HCC
Acetylation (K263)	Post-translational modification	Trafficking switch	Acetylation state influences PD-L1 subcellular distribution between membrane/cytoplasmic and nuclear compartments	BC

*BC* breast cancer, *CC* cervical cancer, *CRC* colorectal cancer, *ESCC* esophageal squamous cell carcinoma, *HCC* hepatocellular carcinoma, *HNSCC* head and neck squamous cell carcinoma, *LUAD* lung adenocarcinoma, *NSCLC* non-small cell lung cancer, *TNBC* triple-negative breast cancer, *UM* uveal melanoma, *CBP* CREB-binding protein, *KPNB1* karyopherin subunit beta 1, *KPNA* karyopherin subunit alpha, *NAT10* N-acetyltransferase 10

^*^Pan-cancer indicates broadly inferred canonical transport relevance not restricted to one tumor type

*Multiple indicates reported across more than one cancer type. Mechanisms summarized here are based on currently available preclinical evidence and may be tumor-context dependent

### Step 2: import licensing & cytoplasmic release

Gate 2 (Nuclear Entry): it governs the transition from endosomal compartments to nuclear import competence. HDAC2-mediated deacetylation of K263 and delactylation of K189 are proposed to relieve membrane-retentive constraints and expose a cNLS-like import interface in the cytoplasmic tail that is compatible with Importin-α recognition, thereby licensing entry into the canonical import pathway [7]. Lactylation at K189, fueled by glycolytic lactyl-CoA, adds a metabolic dimension; its removal by HDAC2 is critical in hepatocellular carcinoma (HCC) for enabling vimentin-assisted import and subsequent pro-tumorigenic gene regulation [57–59]. Genotoxic stressors such as doxorubicin additionally activate p-AKT to promote nuclear translocation and anti-apoptotic programming in breast cancer (BC) [6, 14]. Cofactors including p-STAT3 and vimentin function as context-dependent facilitators that integrate upstream oncogenic signals to bias the PTM equilibrium toward nuclear import in the systems where they are active (Fig. 1, Gate 2; Fig. 2, Step 2) [7, 11, 25, 60]. Rather than acting as obligate transport adaptors, these proteins more likely serve as permissive scaffolds or signal amplifiers that increase the efficiency of importin-α/β1-mediated transport under specific oncogenic or stress conditions.

A candidate upstream mechanism involves ADAM10/17-mediated proteolytic shedding of the PD-L1 extracellular domain, which may relieve glycan-dependent steric constraints and expose the cytoplasmic tail for importin engagement [14, 52]. However, how a membrane-anchored remnant subsequently reaches the nucleus remains unresolved; proposed routes-including lateral diffusion through the ER-nuclear membrane continuum or passage through peripheral NPC channels [61, 62]-currently lack direct experimental support.

Nuclear receptors (NRs) such as TLX, RORC, and PPARγ may modulate the total PD-L1 pool available for nuclear import through transcriptional regulation of CD274 [19, 60, 63]. Because many NRs also rely on importin-α/β1 for their own nuclear entry, competitive interactions with PD-L1 for shared transport machinery are plausible [7, 64]. Targeting NRs may thus reduce nPD-L1 indirectly by limiting PD-L1 production upstream of translocation, rather than by blocking import directly. The regulators active at Gate 2-including HDAC2, lactylation (K189), p-AKT, p-STAT3, vimentin, ADAM10/17, and N-glycosylation (N219)-are detailed in Table 4 [19, 50] (Fig. 1, Gate 2; Fig. 2, Step 2).

### Step 3: importin complex assembly & NPC translocation

The directionality of classical nuclear import is governed by the Ran GTPase cycle. RanGTP, generated in the nucleus by the chromatin-associated guanine nucleotide exchange factor RCC1, is enriched in the nuclear compartment. In the cytoplasm, RanGAP1 promotes GTP hydrolysis, maintaining a low RanGTP state. This well-established gradient-driven mechanism provides the thermodynamic basis for unidirectional nuclear import and is the framework within which PTM-gated PD-L1 nuclear trafficking operates [65].

Following PTM-dependent exposure of a cNLS-like import signal, PD-L1 is proposed to function as an active nuclear import cargo that engages importin-α, which in turn recruits importin-β1 to assemble the classical import complex [7]. Upon entry into the nucleoplasm, RanGTP-enriched in the nuclear compartment-binds importin-β1 and promotes allosteric disassembly of the import complex, thereby releasing PD-L1 into the nucleus [31]. Because the PD-L1 cytoplasmic tail has been proposed to harbor cNLS-like motifs compatible with importin-α recognition, p-STAT3 and vimentin are more likely to act as auxiliary scaffolds or trafficking modulators rather than obligate nuclear import adaptors [7, 14, 66]. Instead, available evidence suggests that these factors may act as context-dependent facilitators that enhance the efficiency of the importin-α/β1 transport pathway. For example, p-STAT3 may help stabilize cargo-associated transport complexes, whereas vimentin may promote perinuclear localization or trafficking of transport-competent PD-L1 [25, 67, 68]. Whether either factor can support alternative import routes independent of importin-α remains unresolved. The transport mediators at this step-importin-α, importin-β1 (KPNB1), RanGTP, p-STAT3, vimentin, and the upstream expression regulator NAT10-are summarized with their proposed roles and cancer contexts in Table 4 (Fig. 2, Step 3).

### Step 4: oncogenic hijacking & context-specific amplification

The following examples show how, in particular genetic contexts, dominant oncogenic pathways appear to hijack this regulatory system to foster adaptive resistance; whether these mechanisms are broadly generalizable remains to be determined [69–71]. In *BRAF* V600E-mutant CRC, constitutive MAPK signaling sustains p-STAT3 activity, which chronically stabilizes the cargo-importin-α/β1 complex to enhance nuclear import efficiency [72, 73]. More comprehensively, in ARID1A-deficient lung adenocarcinoma (LUAD), the loss of this chromatin remodeler triggers a coordinated program: the EZH2/PTEN/E2F1 axis drives PD-L1 transcription, while the MDM2/eIF5B axis boosts its translation [30, 32]. This surge in PD-L1 is coupled with elevated levels of eIF5B and components of the importin-β1 pathway, which exploit deacetylated motifs to execute hyper-efficient nuclear translocation via the canonical importin-α/β1 complex [74]. Inside the nucleus, nPD-L1 forms complexes with CD44 to reactivate Ras signaling, driving resistance to epidermal growth factor receptor** (**EGFR) inhibitors like Osimertinib [30]. Similarly, in cancers with mesenchymal features, vimentin overexpression provides a scaffold that amplifies the nuclear import of deacetylated/delactylated PD-L1, linking translocation to immune-cold phenotypes [12, 34, 75]. The oncogenic signaling components amplifying this process, including ERK/p-STAT3 (CRC), eIF5B/importin-β1 (LUAD), and vimentin (mesenchymal tumors), are catalogued with their respective cancer contexts in Table 4 (Fig. 2, Step 4).

Collectively, PD-L1 nuclear trafficking is best viewed as a multilayered and context-dependent process that links extracellular stress sensing to intracellular survival programs [50]. This framework helps explain how tumors can decouple PD-L1 from its membrane checkpoint role and repurpose it as a nuclear effector of resistance. Therapeutically, each step of the pathway presents an opportunity for intervention, including blockade of licensing PTMs, inhibition of importin-mediated transport, or selective targeting of nPD-L1 itself [13, 16, 32].

### Nuclear PD-L1 as a multifaceted hub: driving tumor malignancy and adaptive evolution

Recent studies suggest that nPD-L1 may function as a context-dependent nuclear regulator linked to transcriptional control, DNA repair, metabolic reprogramming, and inflammatory signalling [1, 7, 14, 50, 76, 77]. We use the term “hub” as a conceptual framework to integrate these observations rather than to imply universally proven causal coordination [11, 24]. This model proposes that tumors may repurpose PD-L1 to convert extracellular stress signals into adaptive cell-intrinsic responses associated with aggressive disease. Below, we summarize evidence for upstream inputs, downstream nuclear functions, and related clinical phenotypes (Tables 2 and  3). Distinct stresses such as genotoxic, inflammatory, metabolic, and oncogenic signals may converge on shared nPD-L1-dependent adaptive programs [60].

Functionally, nPD-L1 has been associated with several stress-adaptive programs [78–80]. In irradiated non-small cell lung cancer (NSCLC) cells, nPD-L1 has been shown to be recruited to double-strand breaks (DSBs), where it bolsters Ku70/80-mediated non-homologous end joining (NHEJ) [14, 79]. NAT10-dependent ac4C modification of importin-β1 mRNA may further sustain nPD-L1 accumulation and cGAS-STING/NF-κB signaling [27, 32]. In HCC, nPD-L1 has been linked to activation of the SQLE-cholesterol pathway [23]. In BC models, chemotherapy-induced nuclear redistribution of PD-L1 has also been associated with GSDMC-mediated pyroptosis and inflammatory escape [6, 34].

For clonal expansion and metastasis, nPD-L1 has been implicated in orchestrating proliferative, migratory, and niche-priming function [12, 31]. It has been shown to drive proliferation by transactivating mitotic regulators like BUB1 in BRAF-mutant CRC or by stabilizing cohesin complexes in BC to ensure genomic fidelity during rapid division [11, 37]. Specifically, in triple-negative breast cancer (TNBC) and other cancers, nPD-L1 was found to integrate into the cohesin complex, compensating for Sororin loss by competing with WAPL for PDS5B binding, thereby regulating chromatid cohesion and preventing multinucleation [35]. It enables dissemination by activating pro-angiogenic programs such as EGR1-VEGF in uveal melanoma (UM) or upregulating stemness factors like TM4SF1 in CRC to promote epithelial-mesenchymal transition (EMT) [11], or interacting with Sp1 to upregulate Gas6 in NSCLC [12]. Additional studies suggest that nPD-L1 may promote an immunosuppressive metastatic niche through recruitment of MDSCs, CAFs, or M2 macrophages [15, 29, 30]. Consistent with these observations, elevated nPD-L1 levels have been associated with poor overall survival or progression-free survival across multiple malignancies (Table 3) [8, 9, 11, 25, 26, 33, 34, 36]. These findings support its potential value as a biomarker of aggressive disease biology.

**Fig. 3:**
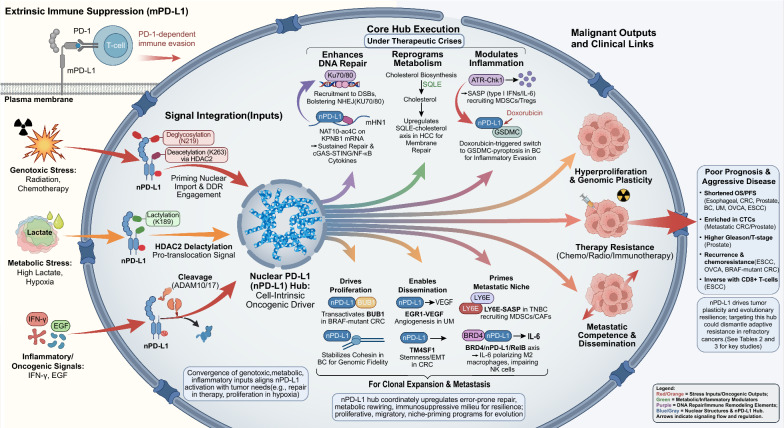
Integration of extrinsic and intrinsic PD-L1 functions as a context-dependent oncogenic hub. Genotoxic, metabolic, inflammatory, and oncogenic cues may promote PD-L1 nuclear accumulation. In this model, membrane-associated PD-L1 mediates immune evasion, whereas nuclear PD-L1 is linked to context-dependent programs involving DNA repair, metabolic rewiring, immune remodeling, and tumor progression. The hub framework is intended to integrate observations from diverse tumor contexts rather than imply a universal mechanism

Overall, current evidence supports a PTM-gated trafficking model in which nPD-L1 may act as a context-dependent nuclear mediator of tumor adaptation. However, most mechanistic data derive from cancer cell lines or genetically engineered mouse models, and whether the proposed sequential gating steps are causally coordinated in vivo remains unestablished. The substantial heterogeneity of reported mechanisms across tumor types further complicates generalization. Future studies employing nuclear-specific perturbation systems, patient-derived models, and dynamic imaging approaches will be essential to determine whether disrupting PD-L1 nuclear trafficking or its downstream transcriptional programs represents a viable therapeutic strategy [31, 35, 77] (Fig. 3).

### The clinical implications and therapeutic strategies of nPD-L1

The discovery of nPD-L1 has prompted a re-evaluation of its canonical role as a membrane-bound immune checkpoint ligand, suggesting potential connections between tumor-intrinsic signaling, immune evasion, and treatment resistance [60, 81]. While definitive clinical validation remains ongoing, a growing body of correlative evidence suggests that nPD-L1 may have emerging clinical relevance through its heterogeneous expression patterns, prognostic associations, and predictive potential [13, 28]. Furthermore, its tractability is being actively investigated as a potential strategy to address treatment resistance to immune checkpoint inhibitors (ICI) and conventional therapies [14]. This section systematically delineates the emerging clinical value of nPD-L1, first examining its potential utility as a candidate predictive biomarker for guiding precision immunotherapy, then analyzing investigational strategies targeting its translocation and function [7, 11, 78], and concluding with a discussion of current challenges and future translational opportunities [27].

**Fig. 4:**
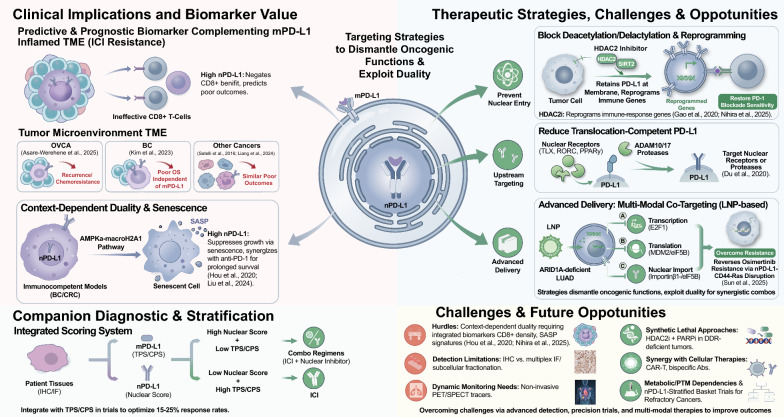
Clinical implications of nuclear PD-L1 and therapeutic strategies for targeting the nPD-L1 hub. High nuclear PD-L1 levels have been correlated with poor prognosis, recurrence, and therapy resistance in retrospective cohorts, although these associations may vary by tumor type and clinical context. Potential therapeutic strategies include inhibition of PD-L1 nuclear trafficking, blockade of associated signaling pathways, and biomarker-guided combination therapy. This figure highlights both established clinical associations and emerging therapeutic opportunities for refractory cancers

A proposed clinical value of nPD-L1 lies in its capacity to complement and refine predictive models based solely on membranous PD-L1 (mPD-L1) [10], particularly in explaining primary ICI resistance within an “inflamed” tumor microenvironment (TME) [34, 82]. One proposed model suggests that nuclear redistribution of PD-L1 may reduce membrane-localized pools while enabling intracellular signaling programs [50, 83]. Clinical observations provide correlative support for this mechanism: high nPD-L1 expression has been reported to negate the survival benefit otherwise conferred by CD8 + T-cell infiltration and to correlate with chemoresistance-linked recurrence-patterns observed across ovarian, breast, and other cancer types (Table 3) (Fig. 4).

The biological role of nPD-L1 demonstrates significant context-dependency, shaped by at least three key variables: TME composition, the PTM state of PD-L1, and the tumor’s genetic background [28, 84]. In immunodeficient or in vitro settings, where immune pressure is absent, the cell-intrinsic oncogenic functions of nPD-L1 dominate unchallenged [35, 81]. Within immune-competent TMEs, however, the immunomodulatory output of nPD-L1 bifurcates into functionally opposing programs. In most contexts, nPD-L1 drives a pro-tumorigenic senescence-associated secretory phenotype (SASP) characterized by IL-6, LY6E, and MDSC/Treg recruitment, fostering immune evasion [31, 34, 75]. By contrast, lactic acid-induced nPD-L1 operating through the AMPKα-macroH2A1 axis can promote cellular senescence and a SASP that tips the balance toward anti-tumor immunity [77, 85]. The molecular switches governing which program is engaged remain unresolved and represent a critical priority for future investigation [86].

This functional dichotomy complicates the straightforward application of nPD-L1 as a biomarker, but also underscores the need for context-aware patient stratification. If the TME and PTM state of nPD-L1 can be systematically characterized, nPD-L1 may serve as a candidate companion biomarker that distinguishes patients likely to benefit from ICI therapy from those in whom nuclear PD-L1 drives resistance [87]. Prospective clinical trials integrating nPD-L1 immunofluorescence scoring with traditional TPS/CPS metrics are needed to test whether this approach can improve upon the 15–25% ICI response rates currently observed across many tumor types [1, 5, 88].

A clinically important but underappreciated implication of the nPD-L1 framework is that standard genotoxic therapies may inadvertently reinforce tumor resilience [8, 14]. Across multiple preclinical contexts, ionizing radiation, doxorubicin, and platinum-based agents have each been linked to distinct mechanisms of stress-induced nuclear redistribution of PD-L1-including HDAC2-dependent deacetylation in NSCLC [29], p-AKT-mediated translocation in BC [89], and nPD-L1-dependent DNA repair engagement in OVCA [22]. These observations converge on a fundamental paradox: the treatments designed to eliminate cancer cells may simultaneously activate adaptive nuclear programs in surviving cells, conferring DNA repair capacity and transcriptional reprogramming that enriches for therapy-resistant clones. This paradox also exposes a structural vulnerability in current immunotherapy strategy. Conventional anti-PD-1/PD-L1 antibodies are designed to block extracellular, membrane-bound PD-L1; they cannot engage the intracellular pool [7]. Consequently, stress-induced nuclear translocation may represent a primary mechanism of adaptive resistance to checkpoint blockade: as PD-L1 redistributes to the nucleus in response to treatment, it escapes antibody targeting while simultaneously acquiring pro-survival transcriptional functions [11, 14]. Under this model, tumors subjected to genotoxic stress may paradoxically become more resistant to ICI precisely because the therapy triggers the nuclear program that antibodies cannot reach.

This mechanistic gap motivates two investigational strategies. First, limiting nuclear accumulation by targeting upstream trafficking regulators-such as HDAC2, importin-dependent transport machinery, or related signaling pathways-has been associated with restored treatment sensitivity and enhanced immunotherapy responses in preclinical models [7, 50, 63]. A second approach is mechanism-based combination therapy [55]. Single-agent checkpoint blockade may be insufficient in tumors with active PD-L1 nuclear trafficking. A dual-targeting strategy-combining conventional anti-PD-1/PD-L1 antibodies with inhibitors of nuclear entry-may therefore be required to simultaneously neutralize membrane-mediated immune suppression and prevent stress-induced transcriptional adaptation in the nucleus [14, 29]. Both approaches remain largely preclinical and require rigorous clinical validation before therapeutic application can be considered.A major barrier to clinical translation of the nPD-L1 concept is the absence of standardized, subcellularly resolved detection methods. Conventional chromogenic immunohistochemistry (IHC) often cannot confidently distinguish nuclear from cytoplasmic PD-L1 staining, limiting the interpretability of existing clinical data [90]. Multiplex immunofluorescence with validated subcellular segmentation, combined with AI-assisted digital pathology, represents a more reproducible approach and should be prioritized for integration into prospective biomarker-stratified trials [91, 92]. Looking further ahead, non-invasive molecular imaging may enable dynamic monitoring of nPD-L1 status during treatment, potentially allowing real-time tracking of therapy-induced nuclear redistribution [93]. Overall, nPD-L1 represents a biologically compelling but clinically early-stage concept. Realizing its translational potential will require three coordinated advances: standardized subcellular detection assays, mechanistically informed patient stratification frameworks, and prospective trials designed to test whether nPD-L1 status predicts differential benefit from checkpoint blockade, nuclear trafficking inhibitors, or dual-targeting combination regimens (Fig. 4).

### Precision targeting of nPD-L1: future directions and opportunities

Although substantial progress has linked nPD-L1 to aggressive disease biology, the field now faces a more fundamental challenge: determining whether nPD-L1 is merely a biomarker of cellular stress or a therapeutically actionable dependency in its own right [11, 77]. Resolving this question has direct implications for drug development-if nPD-L1 is causally required for resistance, intercepting it becomes a therapeutic priority; if it is epiphenomenal, targeting trafficking may offer limited benefit. Most current evidence remains associative, and nuclear-specific perturbation systems are urgently needed to establish causal functions distinct from mPD-L1 [14, 50]. Tools such as endogenous nuclear-localization mutants, compartment-restricted degraders, and inducible trafficking models will be essential to resolve this question [7].

Several mechanistic frontiers warrant immediate attention. First, the intercellular dimension of nPD-L1 biology remains unexplored. Given that tumor cells actively secrete PD-L1 in exosomes, it is conceivable that extracellular PD-L1 may be internalized by neighboring malignant, stromal, or immune cells [68]. Whether such exogenous PD-L1 can access the nuclear compartment via importin-dependent transport and modulate transcriptional or DNA damage response programs in recipient cells is an open question with potential implications for understanding immune evasion at the tissue level [7]. Second, the mitotic fate of nPD-L1 remains unknown [94]. By analogy to epigenetic memory mechanisms, if PTM-licensed nPD-L1 molecules were preferentially retained or rapidly reassembled in daughter cells during mitotic exit, this could establish a form of non-genetic “PTM memory” that enables rapid re-establishment of resistance-associated nuclear programs across cell generations-a mechanism that would be invisible to transcriptomic analyses and resistant to conventional therapeutic targeting [7, 13, 35].

**Fig. 5:**
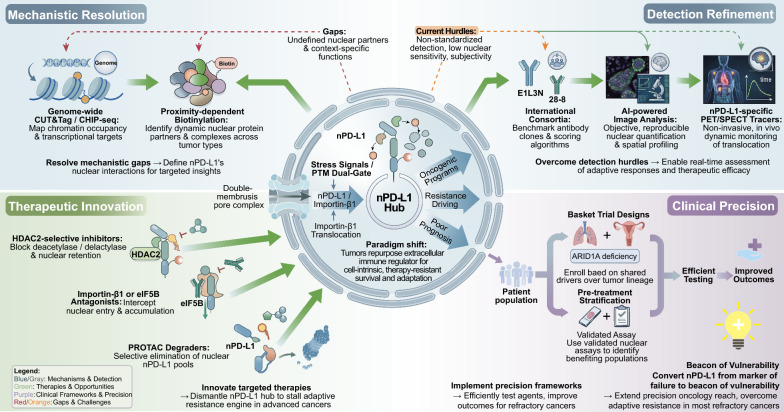
Precision targeting of nuclear PD-L1: future mechanistic, detection, and clinical directions. This mind-map summarizes current gaps and proposed strategies for exploiting nuclear PD-L1 (nPD-L1) as a therapeutic vulnerability. Mechanistic resolution approaches, including proximity labeling, CUT&Tag/ChIP-seq, and biotinylation-based methods, may help define nuclear interactors, chromatin occupancy, and tumor-type-specific complexes. Detection refinement strategies include standardized multiplex imaging, AI-assisted quantification, international benchmarking of antibodies, and the development of nPD-L1-specific PET/SPECT tracers for non-invasive dynamic monitoring. Therapeutic innovation may involve selective HDAC2 inhibitors, blockers of the importin-α/β1 pathway and eIF5B, and nuclear-specific PROTACs to dismantle the adaptive resistance hub. Clinical precision frameworks include biomarker-driven basket trial designs based on shared molecular features such as ARID1A deficiency, pre-treatment stratification, and validated nuclear assays to identify responsive populations. Two speculative but mechanistically grounded extensions are shown as dashed pathways: the intercellular spread of nuclear-import-competent exosomal PD-L1 within the tumor microenvironment, and the mitotic inheritance of PTM-primed nPD-L1, whereby daughter cells may bypass de novo modification requirements and rapidly reconstitute a resistance-competent nuclear program. Overall, the long-term goal is to shift nPD-L1 from a marker of therapeutic failure to a potential beacon of vulnerability in refractory cancers

If nuclear-specific perturbation studies confirm causal roles for nPD-L1, therapeutic efforts should move beyond broad PD-L1 suppression toward selective disruption of nuclear function [7, 34]. Nuclear-restricted PROTAC degraders, import-selective antagonists, and synthetic-lethal combinations with PARP, ATR, CHK1, or metabolic inhibitors may prove especially effective in tumors reliant on nPD-L1-mediated stress adaptation [26, 81]. Critically, such approaches may preserve beneficial membrane checkpoint biology while dismantling cell-intrinsic resistance programs—addressing the mechanistic gap that conventional anti-PD-1/PD-L1 antibodies cannot fill [30]. Clinical translation will require biology-driven trial designs rather than lineage-restricted strategies [83]. Basket or adaptive platform studies enrolling patients according to shared drivers of nPD-L1 activation-such as ARID1A loss [30], mesenchymal transition states, replication stress, or therapy-induced nuclear translocation signatures-may better identify responsive populations. Longitudinal on-treatment sampling will further clarify whether nPD-L1 suppression correlates with durable benefit [13]. Ultimately, the next phase of the field is not simply to measure nPD-L1 more precisely, but to determine whether intercepting nPD-L1 can meaningfully disable tumor plasticity and adaptive resistance in patients (Fig. 5).

## Conclusion

In conclusion, nPD-L1 exemplifies an emerging adaptive principle in oncology: the repurposing of extracellular immune regulators for intrinsic, therapy-resistant survival. The available evidence, while compelling, remains largely correlative and context-dependent. Moving forward, dismantling this nuclear hub represents a frontier in overcoming adaptive resistance, yet definitive causal validation will demand selective manipulation of nPD-L1 without altering its membrane checkpoint function. By systematically mapping its machinery, refining its detection, and innovating targeted therapies, we can aim to convert nPD-L1 from a marker of therapeutic failure into a beacon for vulnerability, ultimately expanding the potential of precision oncology for refractory cancers.

## Data Availability

No datasets were generated or analysed during the current study.

## Abbreviations

ac4C: N4-acetylcytidine
ADAM10/17: A disintegrin and metalloproteinase domain-containing protein 10/17
ARID1A: AT-rich interactive domain-containing protein 1A
ATR: Ataxia telangiectasia and Rad3-related protein
BC: Breast cancer
CBP: CREB-binding protein
cGAS-STING: Cyclic GMP-AMP synthase–Stimulator of interferon genes
Chk1: Checkpoint kinase 1
ChIP-seq: Chromatin immunoprecipitation sequencing
Co-IP: Co-immunoprecipitation
CRC: Colorectal cancer
CSC: Cancer stem cell
CTCs: Circulating tumor cells
cNLS: Classical nuclear localization signal
DSB: Double-strand break
ECD: Extracellular domain
eIF5B: Eukaryotic translation initiation factor 5B
EMT: Epithelial-mesenchymal transition
ESCC: Esophageal squamous cell carcinoma
FG repeats: Phenylalanine-glycine repeats
HCC: Hepatocellular carcinoma
HDAC2: Histone deacetylase 2
HNSCC: Head and neck squamous cell carcinoma
ICB: Immune checkpoint blockade
ICI: Immune checkpoint inhibitor
IF: Immunofluorescence
IHC: Immunohistochemistry
IL-6: Interleukin-6
INM: Inner nuclear membrane
importin-α: Importin subunit alpha (KPNA)
importin-β1: Importin subunit beta-1 (KPNB1)
K189: Lysine residue 189
K263: Lysine residue 263
LUAD: Lung adenocarcinoma
MDSC: Myeloid-derived suppressor cell
MDM2: Mouse double minute 2 homolog
mIF: Multiplex immunofluorescence
NF-κB: Nuclear factor kappa B
NAT10: N-acetyltransferase 10
NHEJ: Non-homologous end joining
NLS: Nuclear localization signal
N219: Asparagine residue 219
NPC: Nuclear pore complex
NSCLC: Non-small cell lung cancer
nPD-L1: Nuclear programmed cell death-ligand 1
OS: Overall survival
OVCA: Ovarian cancer
p300: E1A binding protein p300 (EP300)
PARP: Poly(ADP-ribose) polymerase
PC: Prostate cancer
PD-1: Programmed cell death protein 1
PD-L1: Programmed cell death-ligand 1
PFS: Progression-free survival
PROTAC: Proteolysis-targeting chimera
PTM: Post-translational modification
p-AKT: Phosphorylated protein kinase B
pGSN: Phosphorylated gelsolin
p-STAT3: Phosphorylated signal transducer and activator of transcription 3
RanGAP1: Ran GTPase-activating protein 1
RanGTP: Ras-related nuclear protein bound to GTP
SASP: Senescence-associated secretory phenotype
SQLE: Squalene epoxidase
STAT3: Signal transducer and activator of transcription 3
TIL: Tumor-infiltrating lymphocyte
TME: Tumor microenvironment
TNBC: Triple-negative breast cancer
Treg: Regulatory T cell
TPS: Tumor proportion score
CPS: Combined positive score
UM: Uveal melanoma
WB: Western blot
